# Too much of a good thing: Overproduction of virulence factors impairs cryptococcal pathogenicity

**DOI:** 10.15698/mic2021.05.750

**Published:** 2021-04-20

**Authors:** Julia C. V. Reuwsaat, Tamara L. Doering, Livia Kmetzsch

**Affiliations:** 1 Molecular Biology of Pathogens Laboratory, Biotechnology Center, Universidade Federal do Rio Grande do Sul, Porto Alegre, Brazil.; 2 Department of Molecular Microbiology, Washington University School of Medicine, St. Louis, Missouri, USA.; 3 Department of Molecular Biology and Biotechnology, Universidade Federal do Rio Grande do Sul, Porto Alegre, Brazil.

**Keywords:** capsule, Cryptococcus neoformans, fungal pathogenesis, Pdr802, Titan cells, virulence factors

## Abstract

The regulation of virulence factor production and deployment is crucial for the establishment of microbial infection and subsequent pathogenesis. If these processes are not properly coordinated, the infecting pathogen is less likely to both survive the immune response and cause damage to the host. One key virulence factor of the opportunistic fungal pathogen *Cryptococcus neoformans*, which kills almost 200,000 people each year worldwide, is a polysaccharide capsule that surrounds the cell wall; this structure helps the fungal cells resist engulfment and elimination by host phagocytes. Another important virulence trait is the development of a giant (Titan) cell morphotype that increases fungal resistance to phagocytosis, oxidative stress, and antifungal treatment. We recently identified the transcription factor Pdr802 as essential for *C. neoformans* adaptation to and survival under host conditions both *in vitro* and *in vivo* (Reuwsaat *et al.*, mBio, doi: 10.1128/mBio.03457-20). Cryptococci lacking Pdr802 display enlarged capsules and enhanced Titan cell production, along with dramatically reduced virulence in a mouse model of infection. These results demonstrate that more is not necessarily better when it comes to virulence factors. Instead, precise regulation of these traits, to avoid both under- and overexpression, is critical for the success of this pathogen as it faces the challenges imposed by the host environment.

Cryptococcosis is a fungal disease caused by *Cryptococcus neoformans* and *Cryptococcus gattii*. *C. neoformans* is responsible for the majority of cases in immunocompromised patients, including those responsible for 15% of AIDS-related deaths worldwide. The infection initiates when the host inhales spores or desiccated yeast cells that reach the lungs. In immunocompetent individuals, this invasion is checked by the host immune system, although some cryptococcal cells may remain latent for extended periods. In contrast, in the setting of immunocompromise, this pathogen can establish a pulmonary infection and then disseminate to the central nervous system (CNS), where it causes a frequently lethal meningoencephalitis.

Once inside the host, *C. neoformans* cells display virulence traits that help them survive and proliferate in a harsh environment. These include their ability to grow at mammalian body temperature, production of a polysaccharide capsule and giant (Titan) cells, and secretion of the pigment melanin as well as degradative enzymes such as urease and phospholipase.

The capsule, which surrounds the cell wall, is composed primarily of high molecular weight repeating polysaccharides; similar materials that are shed from the cells impair the host immune response. The capsule changes size and potentially composition depending on the site of infection and helps the fungal cells evade phagocytosis. If cryptococci are nonetheless internalized by host immune cells, capsule material also acts to damage host membranes and protects the yeast against oxidative stress. Cryptococcal cells without capsules are readily phagocytosed and destroyed by macrophages, showing the importance of this structure for the facultative intracellular lifestyle of *C. neoformans*.

Titan cells are a morphotype of *C. neoformans* with a diameter greater than 30 µm (including the polysaccharide capsule). Interestingly, these huge and polyploid cells produce normal-size aneuploid cells during infection. In some cases, this aneuploidy helps the progeny cells survive conditions encountered in the host, such as oxidative stress and antifungal treatment. While their extreme size helps Titan cells to avoid phagocytosis by macrophages, aiding their survival in the lungs, it reduces their ability to cross biological barriers and thus disseminate to the brain.

We recently showed that the transcription factor Pdr802 coordinates multiple aspects of the cryptococcal response and adaptation to the host environment both *in vitro* and *in vivo*. We found that *PDR802* gene expression is highly induced upon growth under host-like conditions (e.g. tissue culture medium at 37°C and 5% CO_2_) and that its deletion adversely affects cryptococcal pathogenicity in a mouse model of infection. A strain in which this gene is deleted displays enlarged capsules and overproduction of Titan cells (**Figure 1**); it further survives poorly in mouse lungs and shows reduced dissemination to the CNS. Overall, this mutant has dramatically reduced virulence compared to wild-type cells.

**Figure 1:**
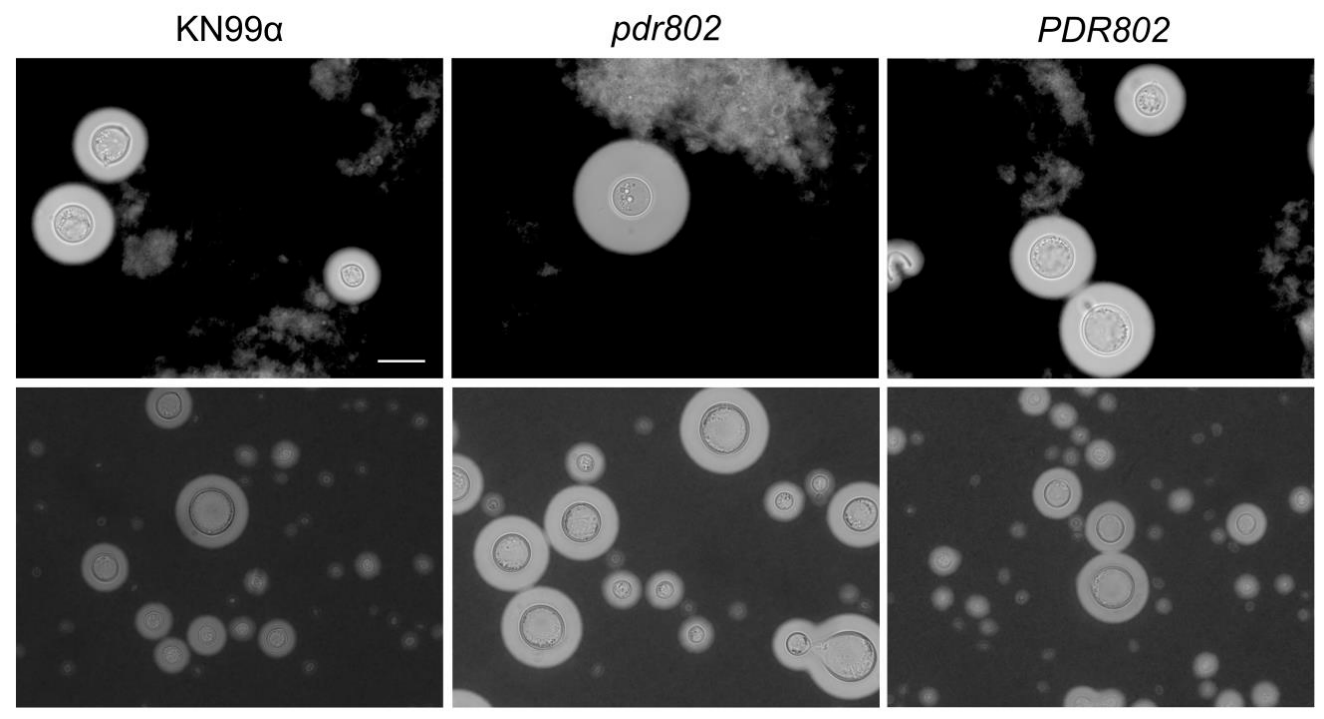
FIGURE 1: The transcription factor Pdr802 is a negative regulator of cryptococcal capsule thickness and Titan cell formation. Upper panel, India ink staining of fungal cells isolated from the lungs of mice infected with the wild-type (KN99α), deleted (*pdr802*), and complemented (*PDR802*) strains at 3 days post-infection. Lower panel, India ink staining of the same strains after growth in Titan cell induction media *in vitro* for 72 hours at 37°C and 5% CO_2_. All images are to the same scale; scale bar, 10 µm.

In pursuing the mechanism of these varied phenotypes, we discovered that Pdr802 regulates multiple genes whose products act in cell wall remodeling, the oxidative stress response, resistance to host temperature, and virulence factor production. For example, Pdr802 directly regulates several genes that influence capsule thickness, such that loss of this transcription factor results in elongated capsule fibers and correspondingly enlarged capsules. Notably, despite this abundance of a known virulence factor, the *pdr802* mutant is significantly less virulent than the wild-type control strain. While some of this change in pathogenicity is likely due to factors beyond the capsule, it may also be influenced by features of the mutant capsule that in some way favor a strong and efficient host immune response.

Like capsule, Titan cells are known to contribute to cryptococcal virulence but are paradoxically overproduced by the hypovirulent *pdr802* mutant. We hypothesize that this is largely due to the inability of *pdr802* cells to properly communicate via quorum sensing, which normally inhibits Titan cell formation, among other functions. In wild-type *C. neoformans*, Pdr802 positively regulates the expression of genes encoding the protease Pqp1, the transporter Opt1, and the transcription factor Liv3, which respectively process, import, and respond to the quorum sensing peptide Qsp1. As a result, cells lacking Pdr802 are unable to sense other cryptococci normally; this impairs their adaptation to the host environment, including modulation of Titan cell formation. This dysregulation, combined with the perturbed expression of other genes that influence Titan cell formation, yields dramatic overproduction of this morphotype.

The examples of capsule and Titan formation highlight the potential danger posed to the pathogen by unmodulated production of even valuable virulence factors. While the inhibition or impairment of virulence factor production generally favors the immune response and host control of pathogens *in vivo*, in these cases an aberrant fungal response to the challenging host environment leads to the failure of cryptococcal infection. Clearly, the balanced regulation of virulence traits is essential for *C. neoformans* to overcome the continuous challenges it encounters in the setting of an infected host (**Figure 2**).

**Figure 2:**
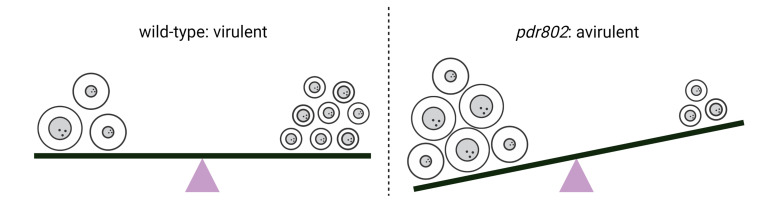
FIGURE 2: Effective pathogenesis requires tight regulation of virulence factors. Optimum levels of capsule thickness and Titan cell formation are key to the success of wild-type infection (left); perturbation of this balance greatly reduces the pathogenicity of the *pdr802* mutant strain (right).

